# A Hypomorphic Lsd1 Allele Results in Heart Development Defects in Mice

**DOI:** 10.1371/journal.pone.0060913

**Published:** 2013-04-24

**Authors:** Thomas B. Nicholson, Anup K. Singh, Hui Su, Sarah Hevi, Jing Wang, Jeff Bajko, Mei Li, Reginald Valdez, Margaret Goetschkes, Paola Capodieci, Joseph Loureiro, Xiaodong Cheng, En Li, Bernd Kinzel, Mark Labow, Taiping Chen

**Affiliations:** 1 Developmental and Molecular Pathways, Novartis Institutes for BioMedical Research, Cambridge, Massachusetts, United States of America; 2 Epigenetics Program, Novartis Institutes for BioMedical Research, Cambridge, Massachusetts, United States of America; 3 Department of Molecular Carcinogenesis, The University of Texas MD Anderson Cancer Center, Smithville, Texas, United States of America; 4 Department of Biochemistry, Emory University, Atlanta, Georgia, United States of America; 5 Developmental and Molecular Pathways, Novartis Institutes for BioMedical Research, Basel, Switzerland; Ludwig-Maximilians-Universität München, Germany

## Abstract

Lysine-specific demethylase 1 (Lsd1/Aof2/Kdm1a), the first enzyme with specific lysine demethylase activity to be described, demethylates histone and non-histone proteins and is essential for mouse embryogenesis. Lsd1 interacts with numerous proteins through several different domains, most notably the tower domain, an extended helical structure that protrudes from the core of the protein. While there is evidence that Lsd1-interacting proteins regulate the activity and specificity of Lsd1, the significance and roles of such interactions in developmental processes remain largely unknown. Here we describe a hypomorphic Lsd1 allele that contains two point mutations in the tower domain, resulting in a protein with reduced interaction with known binding partners and decreased enzymatic activity. Mice homozygous for this allele die perinatally due to heart defects, with the majority of animals suffering from ventricular septal defects. Molecular analyses revealed hyperphosphorylation of E-cadherin in the hearts of mutant animals. These results identify a previously unknown role for Lsd1 in heart development, perhaps partly through the control of E-cadherin phosphorylation.

## Introduction

The development of the mammalian heart is a complex process involving the coordinate interplay of numerous pathways. Because of this, one of the most common causes of lethality in genetically-modified mice involves heart defects [1], and congenital heart defects affect approximately 1% of human newborns [2]. The heart is the first organ to form during development, with differentiation of cardiomyocytes in mice beginning at embryonic day 7.5 (E7.5), leading to the formation of the heart tube. The heart tube begins beating at E8.0, and by E9.0 exhibits a regular beating rhythm [3]. The tube is composed of three layers, the myocardial layer, the endothelial layer, and the cardiac jelly. Recent results have demonstrated that the development of the heart arises from two cell lineages, with the second heart field being the source of the outflow-tract and myocardium [4]. The heart tube loops to the right, leading to the eventual formation of the atria and ventricles with contributions from both the first and second heart fields. In the heart, localized “swellings” of the wall appear at approximately E9.5; these are known as cushions, which arise from the cardiac jelly, and are critical for the formation of all septal and valvular structures of the mature heart [5]. The developing heart is initially composed of a single ventricle; subsequent formation of the septum, separating the ventricle into two distinct compartments, allows for the unidirectional flow of blood through the animal. The muscular portion of the ventricular septum begins to appear at E11.5 [3]. The closure of the ventricles is complete by E14.0, at which point the muscular portion, derived from the heart wall, and the membranous portion, arising from the cushions, have fused to form a complete separation between the ventricles [3].

Cardiac defects are a major source of late-stage lethality during mouse development, including improper formation of the walls between the chambers, outflow tract malformations, and defects in the cardiac conduction system [1]. The genetics underlying these defects are complex, and may involve large numbers of genes [6]; approximately 80% of heart defects in human newborns occur in a sporadic manner, with the genetics still being characterized [7]. Because of the complexity of the development of the heart, and the lack of *in vitro* cellular systems that are available to model its development, the dissection of these pathways has primarily depended on the characterization of mutant mouse models. For example, the initial identification of the second heart field was with a transgenic mouse expressing *lacZ* under the control of the *Fgf10* gene [8]. The proliferation of cardiac progenitor cells appears to be controlled by a complex interaction between Notch1 and β-catenin pathways, and their effects on transcription factors such as Isl1 [9]. Mouse models have since been used to identify many proteins that are involved in the development of the heart (reviewed in reference [4]). Despite this, much about the molecular mechanisms controlling the development of the heart remains to be clarified.

Human lysine-specific demethylase 1 (LSD1, also known as AOF2/KDM1A) was the first of a group of enzymes with lysine-specific demethylase activity to be characterized [10]. LSD1 contains an amine oxidase domain, which demethylates proteins in a FAD-dependent manner, and a Swi3p, RSC8p, and Moira (SWIRM) domain, which is a characteristic of proteins that interact with chromatin. LSD1 exhibits enzymatic activity toward di- and monomethyl histone H3 lysine 4 and lysine 9 (H3K4 and H3K9, respectively); the specificity for H3K9 arises when LSD1 binds to the androgen receptor (AR), resulting in a shift of its activity from H3K4 [11]. This highlights the key role the LSD1 binding partners have in determining its enzymatic targets. The demethylation of H3K4 results in repression of transcriptional activity, while the opposite occurs when H3K9 is demethylated, indicating a context-dependent effect of LSD1 on gene expression. This switch in specificity is aided by phosphorylation of threonine 6 of H3 by protein kinase C β 1, which interacts with the LSD1-AR complex [12]. Several other LSD1 interacting partners have been identified, including the CoREST, CtBP, NRD and BRAF35 complexes as well as Blimp-1 and ZNF217 and ZNF198 [13]–[16]. The interaction of the LSD1/CoREST/HDAC complex with SUMO-2 is important for specific gene repression [17]. Similarly, Myc recruits LSD1 to specific chromatin regions, where it is required for efficient Myc-induced transcription [18]. These interactions occur primarily through the LSD1 tower domain, an insertion in the amine oxidase domain that extends as much as 90Å from the center of the protein.

The activity of LSD1 is not solely directed toward histone proteins. For example, LSD1 demethylates p53 when it is dimethylated at K370 [19]. This results in a loss of p53-53BP1 interaction, leading to a decrease in the promotion of apoptosis by p53, possibly contributing to cancer progression. p53 directly interacts with LSD1, and this interaction serves to promote LSD1 binding to and activity at specific promoters [20]. Demethylation of E2F1 by LSD1 promotes apoptosis by stabilizing the protein, allowing its accumulation through a mechanism involving the inhibition of the ubiquitination of the E2F1 protein [21]. Loss of Lsd1 in mouse embryonic stem cells results in a decrease in Dnmt1 protein levels [22], as methylation of Dnmt1 leads to its degradation. It is likely that further studies will identify other proteins that are the targets of LSD1 action.

We and others have generated Lsd1-null mice and demonstrated that knockout embryos die during the early stages of development [22], [23]. Further studies have begun to elucidate the role of Lsd1 in various organ systems. Expression of Lsd1 is required for neural stem cell proliferation, and knockdown of Lsd1 in the brain results in decreased progenitor proliferation [24]. Interestingly, alternative splicing generates brain-specific Lsd1 isoforms that affect neurite morphology [25], while in rats, upregulation of Lsd1 expression occurs upon brain injury [26]. The interaction of Lsd1 with TAL1, as part of a larger complex, is involved in maintaining erythroid cells in an undifferentiated state [27]. Recent work has also established that Lsd1 is required for adipogenic differentiation, where its activity on methylated H3K9 primes the chromatin for upregulation of key factors involved in adipogenesis [28]. LSD1 appears to play a role in human cancer, as it is overexpressed in tumors from several organs [29], [30]. Conversely, examination of prostate cancer samples indicates minimal expression changes of LSD1 [31]. LSD1 expression is often decreased in human breast cancer, and ectopic expression of this protein is sufficient to decrease metastatic ability [32]. LSD1 interaction with Snai1 is required for repression of epithelial marker genes, and to maintain the epithelial state in cancer cells [33], [34]. In neuroblastomas, LSD1 expression is inversely correlated with the differentiation state of the cells, and knockdown of LSD1 results in defects in cellular growth in xenograft models [35].

While many roles for LSD1 continue to be identified, its *in vivo* role beyond early development remains poorly characterized. This has been hampered in particular by the fact that the Lsd1-null mice die *in utero*. We sought to take advantage of the conditional floxed allele that was generated during the course of our previous study [22] to explore the function of Lsd1 in specific tissues. However, the animals homozygous for the floxed allele exhibited a failure to survive after birth. This suggested that the floxed allele actually represented a hypomorphic allele, and would instead provide further insight into the role of Lsd1 during the later stages of development. The hypomorphic animals suffered from heart defects, identified as the likely cause of perinatal lethality. The floxed allele contained two point mutations, which were subsequently shown to decrease both Lsd1 enzymatic activity and binding to several known interactors. This resulted in the upregulation of a small subset of gene products. In addition, E-cadherin was hyperphosphorylated in these hearts. These results thereby identify a novel role for Lsd1 in the proper development of the mammalian heart.

## Results

### The Floxed Lsd1 Allele is Hypomorphic

**Figure 1:**
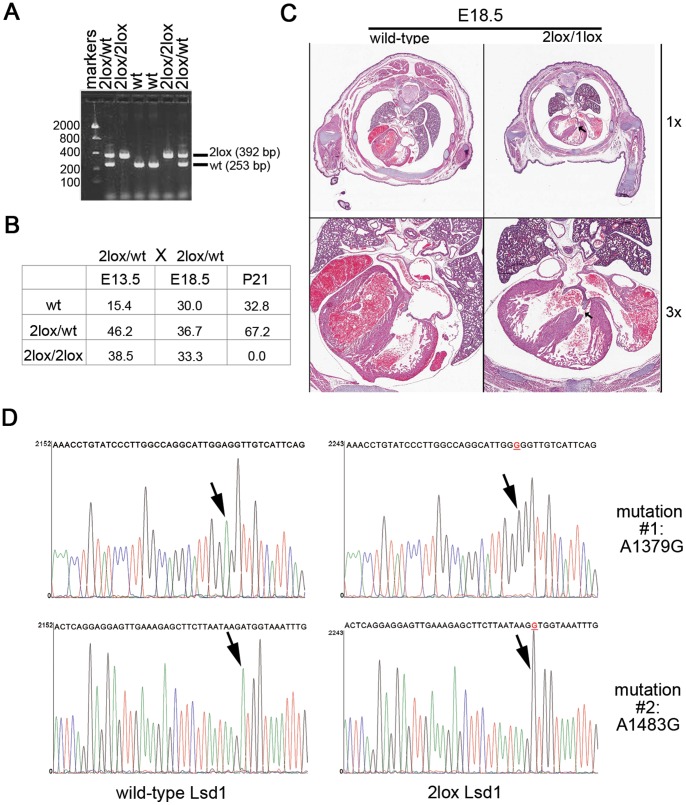
Mice homozygous for the floxed (“2lox”) allele show heart defects. (A) Genotyping of late stage embryos. Genomic DNA was isolated from 6 representative E18.5 embryos, and the *Aof2* allele analyzed by PCR genotyping. Both the wild-type (253 bp) and 2lox (392 bp) alleles can be observed, with two animals homozygous for the 2lox allele. The size, in base pairs, of DNA size standard markers is indicated on the left of the image. (B) Embryos homozygous for the 2lox allele survive to birth, but die shortly thereafter, as shown by the lack of homozygous pups at day P21. The data represents the percent of each genotype present at the indicated time point; for E13.5, this is the total of 3 litters, while E18.5 and P21 represent data from 6 pooled litters each. (C) Representative photomicrograph (H&E staining) illustrating a ventricular septal defect in an E18.5 *Aof2* ^2lox/1lox^ pup at low magnification (upper right hand panel; 1.0x) and higher magnification (lower right hand panel; 3.0x) compared to a wild type control animal (upper and lower left hand panels). (D) Sequence comparison of the wild-type and 2lox *Aof2* alleles. Sequencing results in the regions with the two point mutations are presented for *Aof2^wt^* and *Aof2^2lox^* alleles. The corresponding base pair sequence is written above the respective graphs. The A to G mutations at positions 1379 and 1483 (positions based on mRNA sequence) are highlighted in red, and indicated by the arrows.

Previous reports [22], [23] demonstrated that embryos lacking a functional copy of Lsd1 die early during development, at approximately day E6.5. We therefore attempted to examine the effect of Lsd1 knockout in specific tissues by employing the Cre-lox technology. We sought to generate mice homozygous for the floxed allele (*Aof2* ^2lox/2lox^). Genotyping of late stage embryos demonstrated that homozygous 2lox/2lox animals could reach this stage of development (Figure 1A). However, intercrosses of *Aof2* ^2lox/+^ mice resulted in no *Aof2* ^2lox/2lox^ animals reaching adulthood. A closer examination of the development of these mice indicated that at E13.5 and E18.5 the expected Mendelian ratio was observed (Figure 1B); however, by three weeks of age no *Aof2* ^2lox/2lox^ mice were present. This perinatal lethality is often a sign of a cardiovascular defect, where animals succumb following birth due to the defective circulation of oxygenated blood [1].

Phenotypic analysis of late-stage E18.5 embryos was accomplished by light microscopic evaluation of tissue sections in order to identify any potential developmental abnormalities. Of five E18.5 *Aof2* ^2lox/2lox^ embryos which were completely serially sectioned and tissues subsequently evaluated by light microscopy, three showed ventricular septal defects (VSDs) characterized by incomplete closure of the membranous portion of the ventricular septum (Figure 1C), and the other two showed a left atrio-ventricular valve defect and myodegeneration/mineralization of the heart, respectively. Similar VSDs were also identified in all four *Aof2* ^2lox/1lox^ pups examined (1lox = null allele). For the *Aof2* ^2lox/2lox^ and *Aof2* ^2lox/1lox^ pups which were examined by light microscopy, sectioned tissues from littermate wild-type pups served as appropriate controls. As no major defects in other organs and tissues were observed in *Aof2* ^2lox/2lox^ and *Aof2* ^2lox/1lox^ pups, we concluded that the heart defects were primarily responsible for the perinatal lethal phenotype.

### The Floxed Allele Contains Two Point Mutations

Targeting of *Aof2* generated a conditional allele with floxed exons 10–13 [22]. The loxP sites in the *Aof2* ^2lox^ allele are located in the introns of the gene [22], and are not supposed to affect protein function, so we examined whether the Lsd1 coding sequence was altered during the generation of this allele. cDNA from wild-type and *Aof2* ^2lox/2lox^ mouse embryonic fibroblasts (MEFs) was cloned and the coding sequences compared by sequencing. The sequence of the 2lox allele was found to contain two adenine-to-guanine point mutations (Figure 1D), which had been accidentally introduced in the gene-targeting vector. The mutations resulted in two amino acid changes (E413G and M448V) in the “tower” domain of Lsd1, which is known to mediate protein-protein interactions [36]–[38], and as such could have important negative effects on Lsd1 function. As the crystal structure of Lsd1 has been solved [36], [38], [39], the predicted effect of these point mutations on the structure and function of this protein was then modeled (Figure S1). The amino acid at position 413 is located near the base of the tower domain, and is in close proximity to the catalytic domain. This could potentially affect the enzymatic activity of Lsd1 and/or the structure of the tower domain. The mutation at 448, conversely, occurs at a residue that plays a role in the binding between Lsd1 and CoREST, a protein that is involved in modulating Lsd1 activity [40], and so would be more likely to alter protein-protein interactions. Based on these models, it is therefore possible that the *Aof2* ^2lox/2lox^ mice were affected by decreased protein activity, altered protein-protein interactions, or both.

### Lsd1 Expression during Heart Development

**Figure 2:**
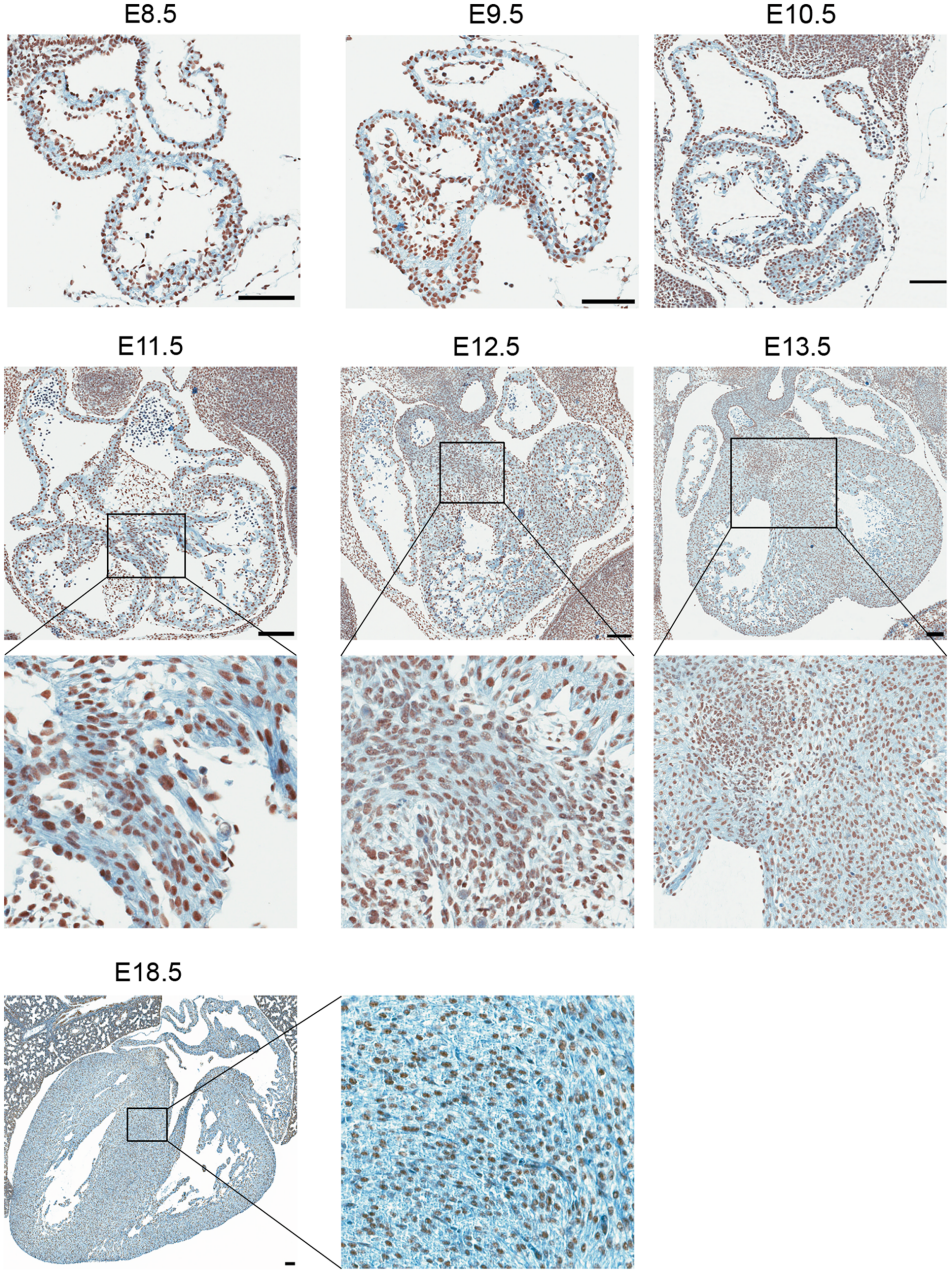
Lsd1 expression in the developing heart. Wild-type embryos at the indicated development stages (E8.5 to E13.5, and E18.5) were dissected and processed for immunohistochemistry. H&E staining was performed, along with staining with anti-Lsd1 to visual the Lsd1 expression pattern during heart development. Note that in all panels, the black bar represents 100 µm.

In order to understand the regulation of Lsd1 expression during development of the heart, wild-type embryos from timed matings were collected at defined timepoints (E8.5– E13.5, and E18.5), and then processed for immunohistochemistry. Staining of heart sections with an anti-Lsd1 antibody revealed widespread expression of Lsd1 in the developing heart (Figure 2). No obvious regions of higher or lower expression, nor any temporal regulation of expression, were observed for Lsd1, suggesting that it has a consistent and ubiquitous expression pattern during the development of the heart.

### The Enzymatic Activity of the 2lox Lsd1 is Altered

**Figure 3:**
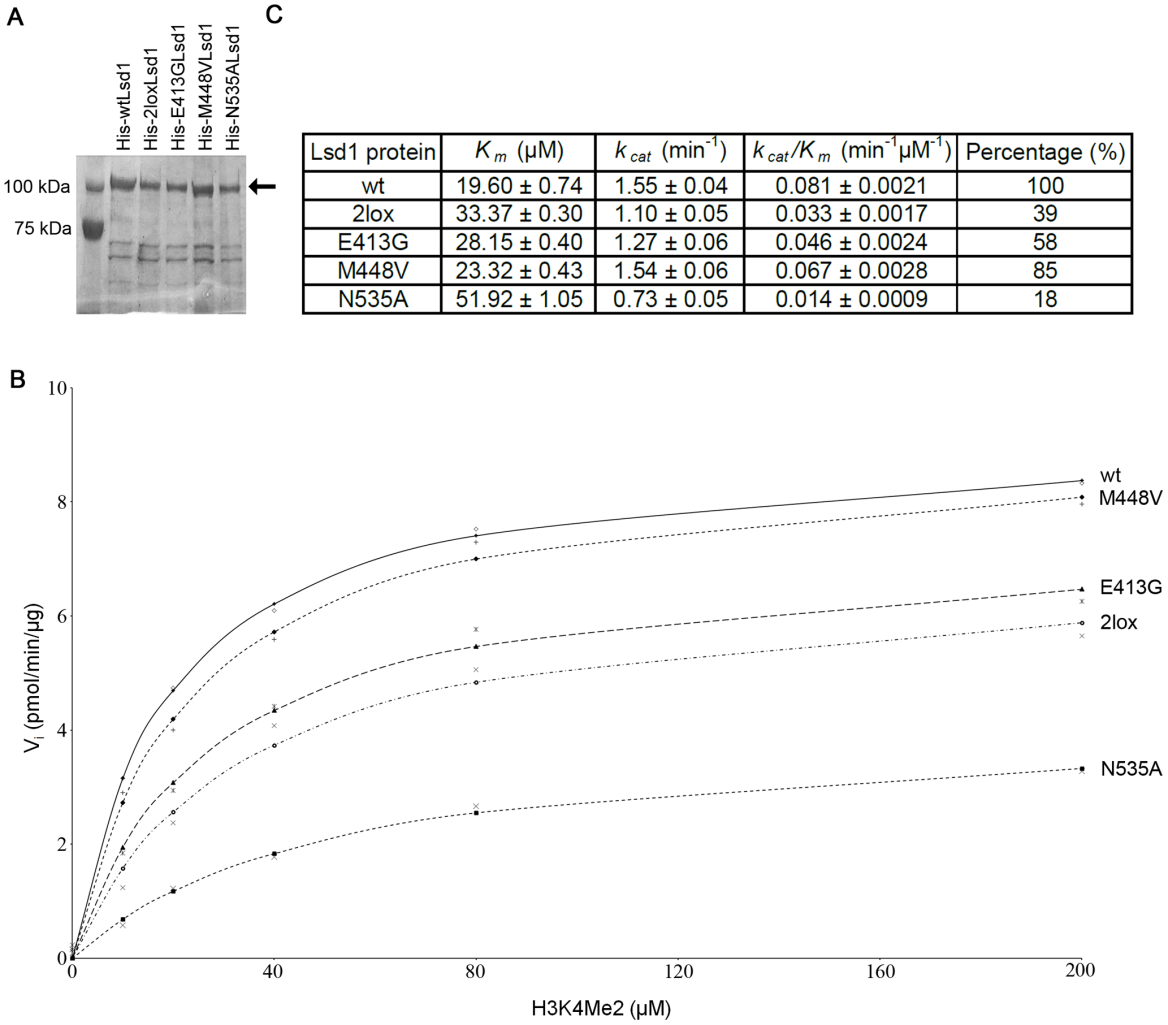
The enzymatic activity of the hypomorphic Lsd1 is reduced. (A) Coomassie blue staining of His-tagged Lsd1 proteins expressed and purified from bacterial cells. The arrow indicates the full-length protein. (B) 10 µg of enzymes were incubated with the indicated concentrations of substrate (H3K4me2) at room temperature. Absorption was measured at 50-sec intervals. Initial rate was obtained from linear least squares fit to the 0–300 sec data and converted to pmol/min/ug by H_2_O_2_ standard. (C) Table demonstrating the *K_m_*, *k_cat_* and *k_cat_*/*K_m_* values for the respective Lsd1 proteins. The percentage value indicates relative activity compared to the wild-type Lsd1.

In order to determine the effect of the point mutations on the enzymatic activity of Lsd1, His-tagged versions of wild-type Lsd1 and the 2lox variant, as well as proteins containing each of the single point mutations present in the 2lox variant (E413G and M448V), were produced in bacteria and purified by affinity chromatography. As well, a point mutant that severely impairs Lsd1 activity (N535A [37]) was generated as a control. Coomassie staining demonstrated the isolation of relatively pure full-length protein for all five variants (Figure 3A). The proteins were then incubated with an H3K4me2 peptide substrate, and the production of the reaction by-product hydrogen peroxide used to monitor the enzyme kinetics (Figure 3B). The *K_m_* and *k_cat_* values were then determined for the respective enzymes (Figure 3C). Using the ratio of *k_cat_/K_m_* it was determined that the efficiency of the 2lox variant was only 39% that of the wild-type enzyme, but considerably higher than that of the N535A mutant (18% of wild-type efficiency). The two single point mutants, E413G and M448V, showed relative activity that was intermediate between the wild-type and 2lox variants; however, the E413G mutant was substantially less active than the M448V mutant (58% vs. 85% of wild-type efficiency). These results demonstrate that the 2lox Lsd1 has reduced demethylase activity, primarily due to the mutation at amino acid 413.

### The Point Mutations in 2lox Lsd1 Affect Protein-protein Interactions

**Figure 4:**
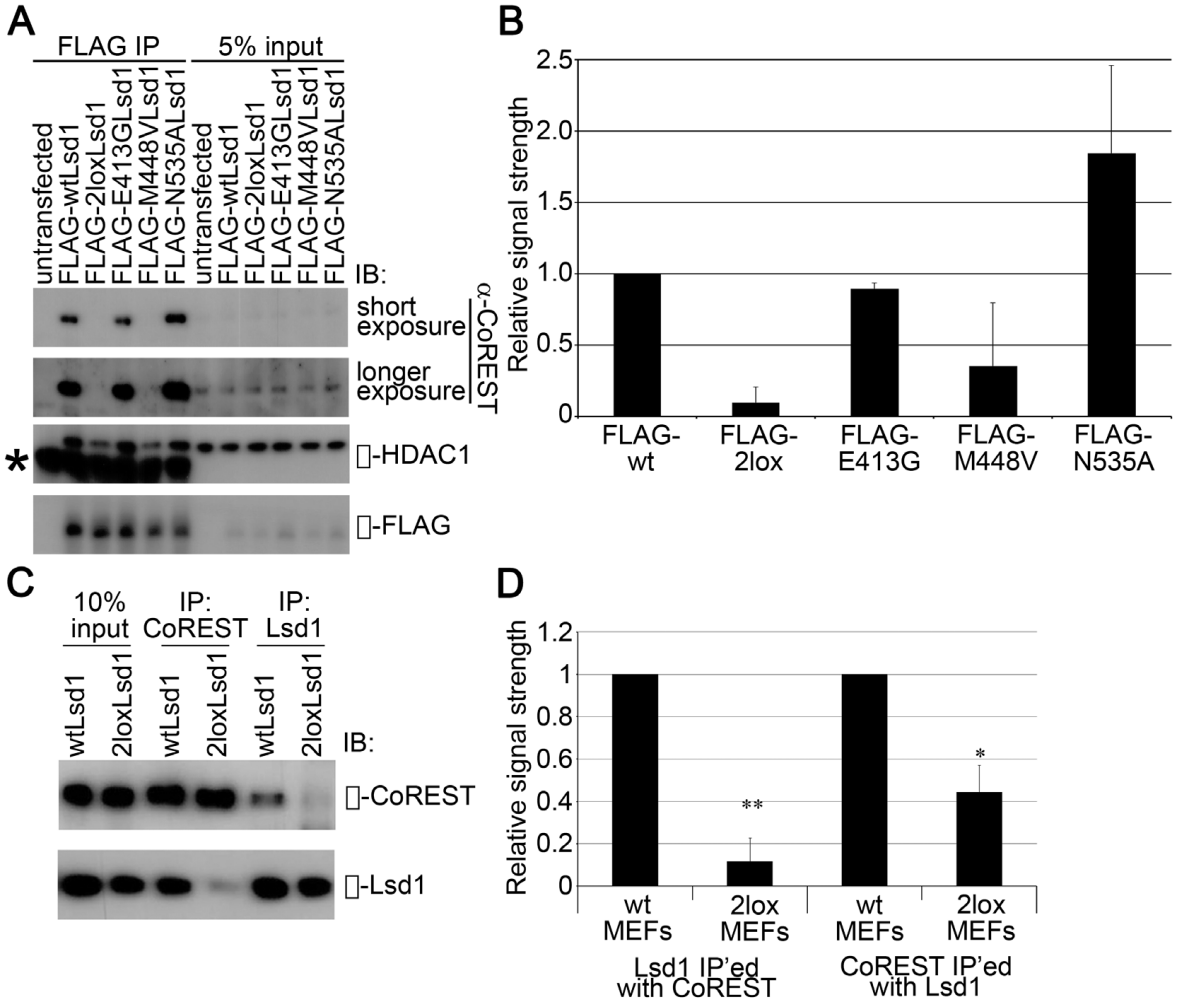
The 2lox Lsd1 variant shows decreased binding to known interactors. (A) Immunoblots of FLAG-based coimmunoprecipitations of NIH 3T3 cells transfected with the Lsd1 variants demonstrate that the 2lox protein exhibits greatly reduced complex formation with known binding partners CoREST and HDAC1. For the two single point mutants, the M448V mutation has a more profound effect on CoREST binding. *: immunoglobulin heavy chain. (B) The relative coimmunoprecipitation of the Lsd1 binding partner CoREST was quantitated using ImageJ software. Data represent the mean +/− SD for two independent experiments. (C) Purification of endogenous Lsd1 complexes from MEF lines confirmed a decrease in Lsd1-CoREST interaction in cells expressing the mutant Lsd1. All westerns are representative of results from experiments performed in triplicate. (D) The decrease in the endogenous interaction of Lsd1 and CoREST by the 2lox variant was quantitated, using ImageJ software. Data represent the mean +/− SD for three independent experiments. **: p<0.001; *: p<0.005, compared to binding by wild-type Lsd1.

Lsd1 interacts with numerous proteins, including CoREST, HDAC1, Zfp198, and the AR [13]–[16]. This could have important consequences for Lsd1 function, as its binding partners determine the activity of Lsd1. For example, Lsd1 demethylates H3K9 only upon interaction with the AR [11]. Because the mutations in the 2loxLsd1 are located in the tower domain, the ability of Lsd1 to bind to known partners was examined. Initially, complexes containing full-length FLAG-tagged Lsd1 variants were isolated from transiently transfected NIH 3T3 cells, and the co-immunoprecipated proteins were identified by immunoblot. The wild-type Lsd1 protein interacted with CoREST and HDAC1, as did the N535A mutant, which demonstrated that the enzymatic activity of Lsd1 is not required for binding (Figure 4A). On the other hand, the 2lox variant of Lsd1 showed substantially reduced binding to both proteins, indicating that the two mutations are sufficient to alter the association of Lsd1 with interacting proteins. This was further demonstrated by quantifying the binding of Lsd1 to CoREST (Figure 4B). The 2lox variant showed a significantly decreased ability to interact with CoREST, compared to both the wild-type and N535A proteins. Examination of the single point mutants demonstrated that the M448V mutation was predominantly involved in the decrease in CoREST binding, as predicted by the structural modeling (Figure S1).

To confirm the results in a more physiologically-relevant system, the interaction between endogenous Lsd1 and CoREST in primary MEFs was examined. Immunoprecipitation of CoREST resulted in more Lsd1 being pulled down in the wild-type MEFs compared to the 2lox/2lox MEFs. Similarly, substantially less CoREST was co-immunoprecipitated with the 2lox Lsd1 compared to the wild-type protein (Figure 4C). Quantification of these results (Figure 4D) confirmed the significant difference in the interaction of Lsd1 with CoREST in these cells. These results indicate that the ability of the Lsd1 2lox variant to interact with its binding partners is compromised, which most likely affects its targeting and function.

### Hypomorphic Hearts Show No Major Cellular Defects

In order to attempt to determine the effect of the hypomorphic Lsd1 allele on heart development at a cellular level, the hypomorphic and wild-type hearts were next compared for proliferation and cellular migration. Pregnant females (E13.5) were injected intraperitoneally with BrdU, and then the embryos harvested one hour later. The hearts from these mice were then examined for proliferation and cardiomyocyte presence. BrdU incorporation in the wild-type and *Aof2^2lox/2lox^* hearts was essentially identical (Figure S2A), indicating that the cardiac defects were not the result of a proliferation deficiency at this stage of development. The defect in ventricular septation may also have resulted from defective cardiomyocyte migration [41]. Therefore, the presence of cardiomyocytes in the developing septum of the embryos was examined to determine if there was a change in cellular population of this structure by staining for sarcomere myosin (Figure S2B). However, no substantial difference in the cardiomyocyte presence was noted between the wild-type and hypomorphic hearts.

### Gene Expression in the Hypomorphic Hearts

Because Lsd1 is known to modify gene expression [10], [11], microarrays were performed on RNA samples isolated from the hearts of wild-type and 2lox/2lox littermates at developmental day E18.5. The data from these microarrays was analyzed to identify gene products that demonstrated significant expression changes between the hypomorphic and control hearts, with the cut-off for significance being an adjusted p-value of 0.05. Using these parameters, only 36 unique gene products demonstrated significant changes between the control and the Lsd1 hypomorphic hearts (Table 1). Consistent with the role of Lsd1 in transcriptional repression, the majority of the genes exhibiting alterations in their expression levels were upregulated in the *Aof2^2lox/2lox^* hearts. Indeed, only 6 gene products were found to be downregulated, including Lsd1 (*Aof2*) itself. Aside from the two point mutations, no unintended genetic alterations were identified in the gene-targeting vector. The reduction of the Lsd1 transcript, therefore, could be due to the insertion of loxP sites or the point mutations, which might affect Lsd1 transcription or processing or mRNA stability. The reduction of Lsd1 probably did not significantly contribute to the heart defects, as mice heterozygous for the Lsd1 null allele (1lox/+), which produce similar amount of Lsd1 as 2lox/2lox mice (Figure S3), showed no obvious phenotype [22].

**Table 1:** Statistical analysis of microarray data (significant probes only).

ID	geneID	Symbol	Alias	logFC	AveExpr	P.Value	adj.P.Val
1423327_at	68172	Rpl39l	4930517K11Rik	1.65661733	4.879415714	4.71E–09	0.000212569
1426762_s_at	99982	Aof2	1810043O07Rik|AA408884|D4Ertd478e|KDM1|LSD1|mKIAA0601	−0.806952025	10.0118442	3.72E–08	0.00051495
1439231_at	-	-	-	1.798007862	4.516676493	4.19E–08	0.00051495
1418744_s_at	57816	Tesc	1010001A17Rik|2410011K10Rik|MGC117906|TE-1	1.324826506	8.587824503	4.57E–08	0.00051495
1417461_at	12331	Cap1	CAP, ADENYLATE CYCLASE-ASSOCIATED PROTEIN 1 (YEAST)	0.940176297	9.858377389	6.21E–08	0.000560536
1426761_at	99982	Aof2	1810043O07Rik|AA408884|D4Ertd478e|KDM1|LSD1|mKIAA0601	−0.830586858	8.667753279	2.34E–07	0.001761303
1424531_a_at	21401	Tcea3	S-II|SII-K1	−1.499887611	6.983186765	2.96E–07	0.001906522
1437678_at	268491	Gm1564	Gm663	1.480513556	4.215117103	3.47E–07	0.001956026
1427242_at	13206	Ddx4	AV206478|Mvh|VASA	2.865609711	5.130828105	4.10E–07	0.001980549
1456515_s_at	277353	Tcfl5	AV260129|Figlb|MGC118431	1.216187744	4.392256828	4.39E–07	0.001980549
1434739_at	207854	Fmr1nb	3830422N12Rik|MGC117577|NY-SAR-35|NYSAR35	2.474837058	6.357752736	5.32E–07	0.002179904
1418743_a_at	57816	Tesc	1010001A17Rik|2410011K10Rik|MGC117906|TE-1	1.071346057	6.682441161	7.10E–07	0.002668349
1436837_at	98558	Mael	4933405K18Rik|AU019877	0.861535783	4.188648361	9.17E–07	0.003182021
1429812_at	69885	2610002D18Rik	MGC169895	−1.010273857	6.400725776	1.35E–06	0.00433516
1449534_at	20962	Sycp3	Cor1|Scp3	0.856261449	4.496058513	1.87E–06	0.005622336
1431393_at	75801	Six6os1	4921504I02Rik|4930447C04Rik|A930035O15Rik|Six6OS	0.630695524	4.480152174	2.28E–06	0.006424947
1449121_at	14105	Fusip1	FUSIP2|NSSR1|NSSR2|Nssr|SRrp40|TASR|TASR1|TASR2	0.762048367	7.914440868	2.92E–06	0.00774473
1438820_at	30054	RNF17	MAX DIMERIZATION PROTEIN MEMBER-INTERACTING PROTEIN 2	1.615141479	5.478954231	3.51E–06	0.008782703
1419004_s_at	12047	Bcl2a1d	A1-d	0.48188309	6.573698785	8.39E–06	0.019920285
1440319_at	329207	Rbm44	Gm817	1.232361133	5.551037194	1.01E–05	0.022836103
1430591_at	13195	DDC	DOPA DECARBOXYLASE	1.120828305	5.575460697	1.14E–05	0.023613271
1423418_at	110196	Fdps	6030492I17Rik|AI256750|Fdpsl1|MGC107162|mKIAA1293	−0.601158533	9.261279901	1.15E–05	0.023613271
1444797_at	382231	8030474K03Rik	Gm1142	1.245922269	4.636070653	1.22E–05	0.023839685
1420641_a_at	59010	Sqrdl	0610039J17Rik|4930557M22Rik	−0.732545714	5.362931986	1.31E–05	0.023839685
1449141_at	74202	Fblim1	2410043F08Rik|Cal|Gt10	0.516562587	8.832495757	1.35E–05	0.023839685
1433707_at	14397	Gabra4	Gabra-4	−0.757299828	5.337890838	1.37E–05	0.023839685
1416889_at	21953	Tnni2	TROPONIN I, SKELETAL, FAST 2	1.324112333	9.537045837	1.47E–05	0.024024794
1426808_at	16854	Lgals3	GBP|L-34|Mac-2|gal3	0.881798344	5.398023447	1.49E–05	0.024024794
1453133_at	73333	Slc25a31	1700034J06Rik|Ant4|Sfec	3.233037918	6.145036273	1.82E–05	0.028026024
1453444_at	70548	5730437C12Rik	RIKEN CDNA 5730437C12 GENE	0.860004042	3.85174898	1.86E–05	0.028026024
1451335_at	231507	Plac8	C15|D5Wsu111e	0.932106891	4.876588334	2.08E–05	0.030318937
1437693_at	110957	D1Pas1	DNA SEGMENT, CHR 1, PASTEUR INSTITUTE 1	0.686257773	4.115476211	2.19E–05	0.030862294
1452730_at	66184	Rps4y2	1110033J19Rik|AA407494|MGC59460	0.584362686	11.13225107	2.37E–05	0.03240169
1447703_x_at	68040	Zfp593	RIKEN CDNA 3110024A21 GENE	−0.803697081	6.564396628	2.44E–05	0.032423765
1455869_at	12323	camk2b	CALCIUM/CALMODULIN-DEPENDENT PROTEIN KINASE II, BETA	1.022786929	6.570458877	2.53E–05	0.032614424
1436778_at	13058	Cybb	C88302|Cgd|Cyd|Nox2|gp91phox	0.830600755	5.023893862	2.76E–05	0.034559966

Legend: ID, Affymetrix probe ID; logFC, log value of the Fold change in expression in the 2lox/2lox hearts; AveExpr, average expression in wild-type hearts;

P.value, unadjusted P-value; adj.P.Val, adjusted P-value.

**Figure 5:**
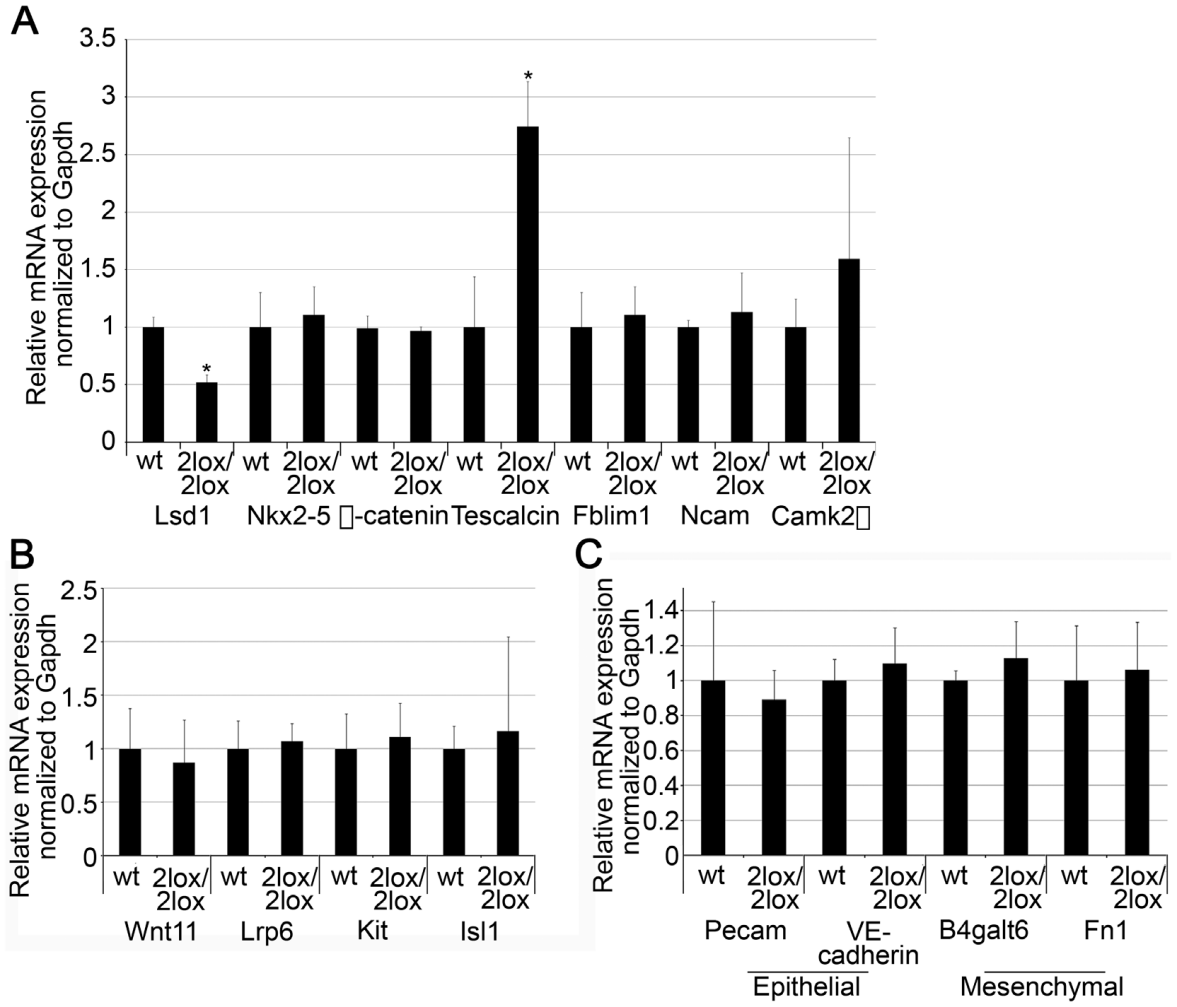
**Changes in gene expression in the hypomorphic hearts.** Expression of gene products, examined by qRT-PCR using RNA isolated from E18.5 embryonic hearts. Data represents mean +/− SD from 3 to 5 animals of each genotype, and was normalized such that the expression of the mRNA in wild-type animals was equal to 1 (*: p<0.05). (A) Lsd1 mRNA levels were decreased by approximately 50%, confirming the microarray data. Nkx2-5, a heart marker, Ncam, and β-catenin were not found to be significantly altered in the hypomorphic hearts. The expression of Tescalcin and Fblim1, two proteins with potential roles in heart development that were identified by microarray, was also analyzed. The increase in Tescalcin expression was recapitulated by the qRT-PCR, while no difference in Fblim1 expression between the wild-type and hypomorphic hearts was noted. CamK2β showed minor increases in expression. (B) Expression of Wnt targets in the wild-type and hypomorphic hearts. The Wnt signaling pathway target genes Wnt11, Lrp6, Kit, and Isl1 were examined for changes in mRNA levels in the hypomorphic hearts by qRT-PCR. No statistically significant changes were noted in the expression of any of these genes. (C) mRNA expression of epithelial and mesenchymal markers is identical between wild-type and 2lox/2lox hearts. Expression of the epithelial markers Pecam and VE-Cadherin, and the mesenchymal markers B4galt6 and Fn1 was examined by qRT-PCR using RNA isolated from E18.5 embryonic hearts. No statistically significant difference was noted in the expression of any of these mRNAs.

In order to confirm these results, RNA samples were isolated from hearts of E18.5 embryos for quantitative real-time polymerase chain reaction (qRT-PCR) analysis. The hypomorphic hearts showed increased expression of Calcium/calmodulin-dependent protein kinase 2 beta isoform (*CamK2β*) compared to wild-type hearts, although the extent of the increase varied greatly between 2lox/2lox animals and was not statistically significant (Figure 5A). The difference in mRNA levels of Fblim1, as observed by microarray, was not recapitulated in this case, while the increase in Tescalcin was confirmed (Figure 5A). Tescalcin, which encodes an EF-hand calcium-binding protein, is highly expressed in the heart, but its role, if any, in heart development is largely unknown. Several genes known to be involved in heart development - Nkx2-5, β-catenin, and Ncam – were also analyzed. None of them showed obvious alterations in the hypomorphic hearts (Figure 5A). Consistent with the microarray data, mRNA levels of Lsd1 were decreased by approximately 50% (Figure 5A).

Despite the lack of Wnt pathway activation noted in the microarray results (Table 1), alterations in Wnt pathway targets were examined due to the important role this pathway has during cardiac development [42]. Thus, the mRNA expression of Wnt11, low density lipoprotein receptor-related protein 6 (Lrp6), Kit, and Islet1 (Isl1), all of which are known to be targets of the Wnt pathway, was compared between the wild-type and hypomorphic animals. No major difference was noted in the expression of any of the genes (Figure 5B), confirming the microarray results.

Lsd1 interacts with Snai1, which plays a role in the regulation of epithelial-mesenchymal transition (EMT) [33]. Because the formation of a complete ventricular septum requires, in part, that cells undergo an EMT, we examined the expression of epithelial and mesenchymal markers in the hypomorphic hearts. The expression of Pecam1 and VE-cadherin, two epithelial markers, and of UDP-Gal:betaGlcNAc beta 1,4-galactosyltransferase, polypeptide 6 (B4galt6) and Fibronectin1 (Fn1), two mesenchymal markers, was thus determined by qRT-PCR. No significant change in expression was noted for any of these RNAs (Figure 5C), suggesting that EMT was not affected in the defective hearts.

### Increased E-cadherin Phosphorylation in the Hypomorphic Hearts

**Figure 6:**
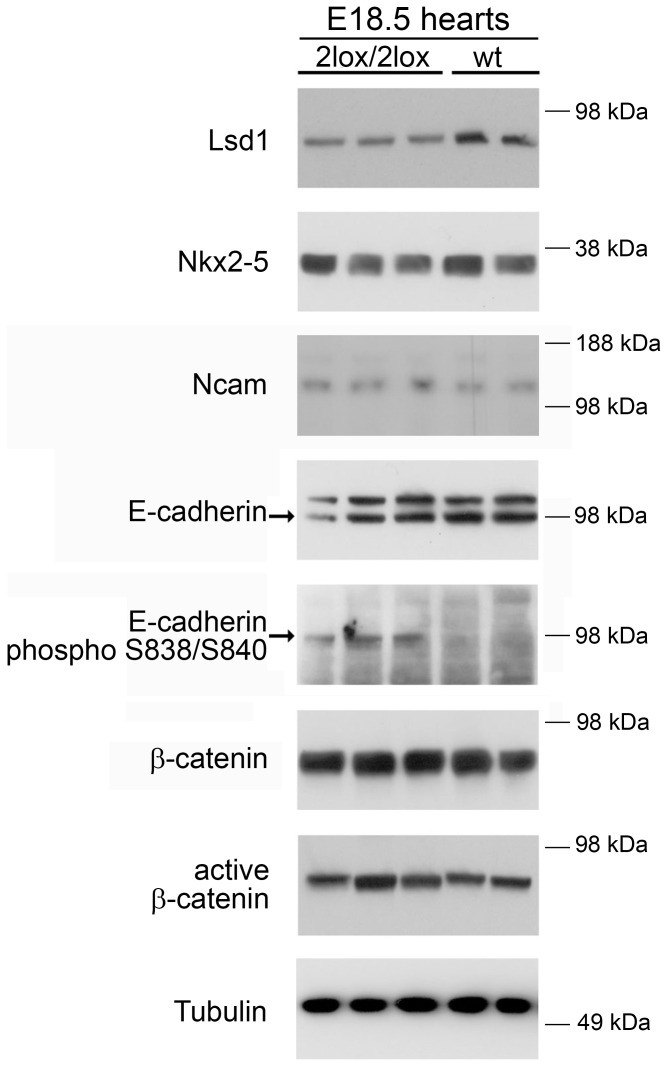
Protein expression in the developmentally-defective hearts. The expression of proteins in 3 hypomorphic and 2 wild-type control E18.5 hearts was examined by immunoblotting. A decrease in Lsd1 and a major increase in phosphorylation of E-cadherin were noted in the *Aof2* ^2lox/2lox^ hearts, whereas all other proteins examined showed no obvious changes. In the E-cadherin blot, the arrow indicates the correct E-cadherin band, and the identity of the higher band is unknown. The Ncam antibody recognizes the 140 kDa isoform of this protein. Relevant molecular weight markers are indicated, in kDa, to the right of each panel.

Because none of the genes identified by microarray were obvious causes of the cardiac defects, a candidate approach was employed to examine pathways involved in heart development. Lysates were generated from E18.5 hearts and subjected to immunoblotting (Figure 6). In agreement with the microarray and qRT-PCR results, Lsd1 showed an approximately 50% reduction in the hypomorphic hearts, whereas Nkx2-5, an important transcription factor involved in heart development, was not altered. While the expression levels of Ncam and E-cadherin, adhesion molecules involved in heart development, did not appear to be substantially different between the wild-type and hypomorphic hearts, the phosphorylation of E-cadherin (on serines 838 and 840) was significantly increased in hypomorphic hearts. We also examined the levels of active and total β-catenin and observed no obvious difference between wild-type and 2lox/2lox hearts (Figure 6).

**Figure 7:**
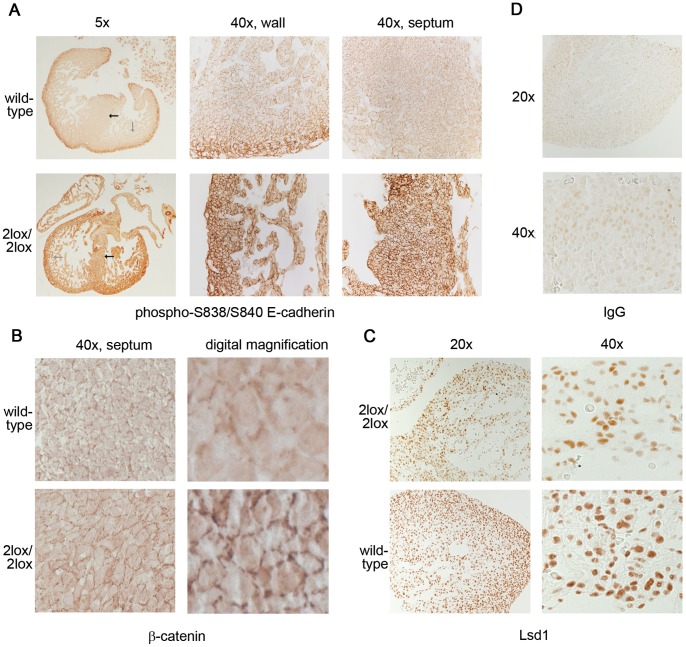
Immunohistochemistry of the hypomorphic hearts. (A) Staining with an antibody specific for the phosphorylation of E-cadherin is strongly increased in the *Aof2* ^2lox/2lox^ hearts compared to the control. The arrows indicate the regions (grey for heart wall, black for septum) from which the higher magnification images (on right) originate. (B) β-catenin localization is altered, with more of the protein present at the plasma membrane in *Aof2^2lox/2lox^* hearts. (C) Lsd1 staining shows slightly decreased signals in *Aof2^2lox/2lox^* hearts. (D) Staining with a non-specific IgG control antibody confirms the specificity of the staining. To minimize background, no counterstain was used. All photomicrographs constitute representative fields; magnification factor is provided above or beside the photographs.

In order to confirm these results, we carried out immunohistochemistry on the E18.5 wild-type and hypomorphic hearts. In agreement with the immunoblotting data, there was a general, major increase in the phosphorylated form of E-cadherin in the 2lox/2lox hearts compared to the wild-type animals (Figure 7A). Phosphorylation of E-cadherin has been shown to enhance its affinity to bind β-catenin [43], [44]. Indeed, the localization of β-catenin appeared altered in 2lox/2lox hearts, with a greater proportion of the protein localized to the plasma membrane and a lower amount in the cytoplasm (Figure 7B). The levels and localization of other proteins examined, including Notch1, total E-cadherin, Nkx2-5, and VEGF, showed no obvious differences between wild-type and hypomorphic hearts (data not shown). Expression of Lsd1 was similar in these animals, with a slightly decreased strength of staining in the 2lox/2lox hearts (Figure 7C). Staining with a non-specific IgG control antibody confirmed the specificity of the staining (Figure 7D). Together, our results suggest that Lsd1 plays a role in controlling the balance of phosphorylation of E-cadherin in the heart.

## Discussion

In this study, we have identified a previously unknown role for the lysine demethylase Lsd1 in cardiac development in mice. Previous reports examining Lsd1 function *in vivo* employed knockout mice that showed early embryonic lethality (at approximately E6.5) [22], [23], thereby precluding an examination of the contribution of Lsd1 to later stages of development. We have characterized mice homozygous for an *Aof2* allele that encodes a hypomorphic protein containing two point mutations in the tower domain. This Lsd1 variant exhibits reduced demethylase activity (Figure 3) as well as decreased binding to known interacting partners (Figure 4), although it does retain residual amounts of both activities. The resulting mice demonstrated cardiac development defects, primarily in the form of VSDs (Figure 1). This study therefore establishes a role for Lsd1 during the development of the mammalian heart.

Expression analysis of the hypomorphic Lsd1 hearts demonstrated that only a small subset of gene products showed altered expression in these hearts (Table 1). While Lsd1 has been shown to play a role in both activation and repression of specific genes, depending on the context [23], overexpression of this protein does not result in an appreciable global decrease in H3K4 methylation (data not shown), in contrast to the closely related Aof1/Lsd2/Kdm1b [45]. This would appear to indicate that Lsd1 activity is selective for specific promoters, and may explain the minor gene expression alterations noted. None of the genes identified by microarray are known to be critically involved in heart development, although reports have indirectly implicated some of the proteins. For example, Tescalcin is expressed in the developing heart [46], [47] and modulates the function of calcineurin and the Na+/H+ exchanger Nhe1 [47]–[49]. Because Nhe1 [48] is a protein that plays an important role in the differentiation of cardiomyocytes [50], it is tempting to postulate that the defect noted arises due to the overexpression of Tescalcin. However, inhibition of Nhe1 results in decreased levels of several important cardiac transcription factors, including Nkx2-5 [50]. Our results demonstrated no alteration in the expression of Nkx2-5 at either the mRNA or protein levels (Figures 5A and 6), arguing against altered Nhe1 activity in the hypomorphic hearts. It is possible, however, that Tescalcin acts in an Nhe1-independent pathway. Further work is required to determine the significance of Tescalcin upregulation in the heart defects noted in the Lsd1 hypomorphic mice.

Because of the lack of promising proteins identified by microarray, we undertook a candidate approach, whereby pathways known to be involved in cardiac development were analyzed by immunoblotting and immunohistochemistry for alterations in the hypomorphic hearts. Among the molecules examined was E-cadherin, which not only has a role in heart development [51], but is a target of Lsd1-mediated transcriptional repression [33]. Total E-cadherin levels were essentially unchanged in the heart, but a major increase in phosphorylated E-cadherin was noted (Figures 6 and 7), suggesting that while the overall levels of this protein are not altered its functional state is different.

Cell adhesion is closely regulated in the heart [52]. For example, Ncam is expressed in a specific manner during chick heart development [53]. Expression of the tenascin variants TNC and TNX, which are important for tissue remodeling, also varies greatly during heart development [54]. N-cadherin is essential for embryonic development, with knockout animals showing heart defects that can be partially overcome by reexpressing this protein specifically in muscles [55]. The altered phosphorylation status of E-cadherin in the hearts may affect cell-cell adhesion in this organ. Phosphorylation of E-cadherin results in significantly enhanced binding to β-catenin, by as much as 300 fold [43], [44]. In breast cancer cells, phosphorylation of β-catenin by the kinase CKIα results in the increased formation of E-cadherin/β-catenin complexes and increases intercellular adhesion [56]. Thus, the increased phosphorylation of E-cadherin in the Lsd1 hypomorphic hearts likely results in strengthened intercellular adhesion. Immunohistochemistry analysis of the hypomorphic hearts demonstrated an enrichment of β-catenin at the plasma membrane (Figure 7B), consistent with this model. Phosphorylation of E-cadherin is mediated, *in vitro*, by both GSK3β and casein kinase 2 (CK2) [44], [57]. qRT-PCR analysis revealed no difference in CK2 expression levels, while GSK3β levels showed a mild decrease (data not shown). It will be interesting to determine if Lsd1-mediated demethylation of a non-histone target is important for this hyperphosphorylation. Phosphorylation of E-cadherin affects both localization and function of this adhesion molecule [58], ultimately leading to an increase in its adhesive properties [57]. Although we did not notice a significant change in cardiomyocyte migration into the septum (Figure S2), it is possible that the increased adhesiveness of the phosphorylated E-cadherin interferes with the final closure of the septum of the heart in the hypomorphic animals.

Other possible mechanisms by which the Lsd1 hypomorphic allele leads to cardiac defects can be envisaged. For example, alterations in EMT may underlie some of the defects, including VSDs, given that Lsd1 plays an important role in the modulation of EMT [33] and that the development of the ventricular septum and heart valves relies on EMT of endocardial cells. However, several lines of evidence argue against a major defect in EMT in Lsd1 hypomorphic animals. First, 2lox/2lox pups were able to develop to term without major defects in other organs and tissues, suggesting that pathways involved in EMT were largely unperturbed. Second, examination of the expression of epithelial and mesenchymal markers in the hypomorphic hearts demonstrated no appreciable difference compared to wild-type hearts (Figure 5C). Finally, total E-cadherin protein level was not altered in the hypomorphic hearts (Figure 6), indicating that the repressive activity of Snai1 on E-cadherin, which requires Lsd1, was not affected. Nevertheless, we cannot exclude the possibility that subtle changes in EMT may have contributed to the heart defects noted in Lsd1 hypomorphic mice.

Defects in cardiac development due to changes in gene dosage are becoming a common observation. For example, insufficiency in the TAB2 gene results in congenital heart defects in humans and zebrafish [59]. In mice, the loss of a single allele of the critical cardiac transcription factor Nkx2-5 results in heart development defects [60]. Tbx5 insufficiency alters the expression of genes critical for cardiac development and leads to proliferation and migration defects [61]. The cardiac transcription factors Hand1 and Hand2 also show gene dosage effects on cardiac development, with decreased expression leading notably to ventricular abnormalities [62]. Because the expression of developmental genes must be maintained in the correct spatio-temporal pattern, it would seem likely that epigenetic factors will play an important role in regulating this process. Indeed, the role of epigenetic factors in heart development has gradually been coming to light. BAF60C, a member of the BAF chromatin remodeling complex, serves as a bridge linking cardiac transcription factors, including Gata4, to this complex [63]. A partial loss of BAF60C is sufficient to affect outflow tract development; complete knockout results in even greater defects. Similarly, the deletion of the lysine methyltransferase BOP results in defects in cardiomyocyte differentiation and development of the right ventricle [64]. Cardiac-specific disruption of the H3K79 methyltransferase Dot1L results in chamber dilation, increased cardiomyocyte cell death, systolic dysfunction, and conduction abnormalities [65]. Whole-body deletion of HDAC2 results in perinatal lethality due to a range of heart defects, while heart-specific deletion of HDAC1 and HDAC2 also results in malformed hearts and death at birth [66]. This study provides the first evidence that Lsd1 plays a key role in the final stages of heart development in the mammalian embryo.

In summary, we have identified a role for the lysine demethylase Lsd1 in the development of the mammalian heart, potentially through effects on the phosphorylation of E-cadherin. Mice containing a hypomorphic variant of Lsd1 demonstrate a highly penetrant defect in the formation of the septum separating the ventricles. This is despite only a small number of genes that are misregulated in the heart. Hyperphosphorylation of E-cadherin may be sufficient to interfere with migration of the cells, leading to the formation of VSDs. VSDs are common congenital defects in human infants, and future work examining the status of LSD1 in children with heart development defects will clarify the role of this protein in human cardiac malformation.

## Materials and Methods

### Animal Use Ethics Statement

The mice used in these experiments, and the generation of the *Aof2* floxed allele, have been previously described [22]. All animal procedures employed in this study were approved by the Novartis Institutes for BioMedical Research Institutional Animal Care and Use Committee (Protocol Number: 09 DMP 053). Genotyping of the animals was performed using primers 440 (5'- CAG TGA TGT ATA CCT CTC ATC AAG -3') and 441 (5'- TAC AGA TTT CAC TGT AAG CAT ATG -3'). The resulting bands are 392 bp for the 2lox allele, and 253 bp for the wild-type allele.

### Antibodies

Antibodies used in this study were: rabbit anti-LSD1 (Cell Signaling, 2139), mouse anti-Tubulin (Calbiochem, CP06), mouse anti-FLAG M2 (Sigma, F 1804), mouse anti-active-β-catenin (Millipore, 05-665), rabbit anti-phosphoS838+S840 E-cadherin (Abcam, ab76319), rabbit anti E-cadherin (Abcam, ab53033), mouse anti-sarcomere myosin (Developmental Studies Hybridoma Bank, University of Iowa, MF20), mouse anti-HDAC1 (Cell Signaling Technology #5356), rabbit anti-CoREST (Abcam, ab32631), rabbit anti-β-catenin (Abcam, ab6302), rabbit anti-NCAM (Cell Signaling Technology #3606), rabbit anti-monomethyl-Histone H3(Lys4) (Millipore, 07-436), rabbit anti-dimethyl-Histone H3(Lys4) (Millipore, 07-030), rabbit anti-Histone H3 (Millipore, 07-690). Immunoblotting and immunohistochemistry procedures were carried out employing standard protocols and antibodies at the manufacturer’s recommended dilutions; chemiluminescence of immunoblots was developed using ECL-plus (GE Life Sciences). In all immunohistochemistry experiments a negative control was included, consisting of a non-specific rabbit IgG antibody, to ensure the specificity of the staining (an example is shown in Figure 7D).

### Histopathology

To morphologically phenotype hypomorphic animals by light microscopy, embryos at known developmental stages from *Aof2^2lox/+^* intercrosses were dissected out of deciduas, and fixed in 10% neutral buffered formalin for 24 hours. Samples were subsequently routinely processed, embedded in paraffin, and serially sectioned at 5.0 µm. Tissue sections were routinely stained with hematoxylin and eosin (H&E), and then examined by bright field light microscopy by a board certified veterinary pathologist for any potential morphological abnormalities.

### Lsd1 Cloning and Mutagenesis

Lsd1 wild-type and 2lox cDNA was generated by isolating RNA from primary mouse embryonic fibroblast (MEF) cell lines homozygous for the respective Lsd1 allele. Total cellular RNA was converted to cDNA using SuperScript reverse transcriptase (Invitrogen) and an oligo dT primer, and then the Lsd1 sequence amplified using high-fidelity KOD polymerase (Novagen) and specific primers (see Table S1). The amplified cDNAs were cloned into the *EcoR*I-*Kpn*I sites of the p3XFLAG-myc-CMV-26 vector (Sigma-Aldrich) to generate FLAG-wtLsd1 and FLAG-2loxLsd1 (Note that the C-terminal myc tag would not be part of the protein because of the presence of a stop codon in the Lsd1 cDNA). These constructs were sequenced on both the template and complementary strands, in duplicate, to identify point mutations in the 2lox coding sequence. Site-directed mutagenesis to generate single point mutants employed the primers described in Table S1 and the QuikChange Site-Directed Mutagenesis Kit (Stratagene). The His-tagged Lsd1 constructs were generated by subcloning the *EcoR*I-*Sal*I fragments of the corresponding FLAG-tagged constructs into pET-28b(+) (*EcoR*I partial digestion was used for the N535A construct, because an internal *EcoR*I site was introduced in the FLAG-N535ALsd1 construct). All constructs were verified by sequencing.

### Immunoprecipitations

NIH 3T3 cells were transiently transfected using Lipofectamine 2000 (Invitrogen) with vectors expressing FLAG-tagged Lsd1 variants. 48 hours later, the cells were lysed with FLAG complex buffer (10 mM Tris, pH 8.0, 140 NaCl, 1.5 mM MgCl_2_, 1 mM DTT, 0.5% Nonidet P-40, 0.1 mM NaVO_4_, 10 mM NaF, 10 mM glycerol phosphate, 1× protease inhibitor cocktail), and the lysate incubated with anti-FLAG resin (Sigma-Aldrich) for 3 hours at 4°C. The beads were subsequently washed 4 times with lysis buffer, and then resuspended in reducing SDS-PAGE sample buffer and analyzed by immunoblot. For immunoprecipitation of endogenous complexes, 90% confluent MEF lines were lysed with the same lysis buffer, and pre-cleared with Protein A/G Plus beads (Santa Cruz Biotechnology) for 1 hour at 4°C. Samples were then left overnight with primary antibody (1∶100 dilution). Protein A/G Plus beads were added to the samples, which were left shaking at 4°C for 2 hours. The beads were then washed 4 times with lysis buffer and resuspended in SDS-PAGE sample buffer for immunoblotting. For changes in immunoblot intensity, films containing bands in the linear exposure range were scanned and analyzed by the ImageJ program (http://rsbweb.nih.gov/ij) for relative density measurements. Statistical significance was determined using the *t*-test (http://www.physics.csbsju.edu/stats/t-test_bulk_form.html).

### *In vitro* Demethylase Assays

Wild-type and mutant Lsd1 recombinant proteins with an N-terminal 6xHis tag were produced in E. coli and purified with nickel affinity chromatography using standard protocols. The demethylase activity of these proteins was tested using the LSD1 Fluorimetric Drug Discovery Kit (Enzo Life Sciences), following the manufacturer’s instructions. Briefly, 10 µg of each Lsd1 protein (wild-type or mutant) were incubated with H3K4me2 peptide at various concentrations (0–200 µM) at room temperature, and the concentrations of hydrogen peroxide (H_2_O_2_), the reaction product, were measured at 50-second intervals for 300 seconds. Km and Vmax values were obtained from a direct least-squares fit to the Michaelis-Menten equation.

### RNA Expression Analysis

Total RNA samples were isolated from freshly dissected E18.5 hearts using an RNeasy Plus kit (QIAGEN). RNA was then hybridized to Affymetrix Mouse430 GeneChip. Analysis of the results from the microarrays was performed as previously described [67]. Briefly, statistical significance was determined using false discovery rate corrected p-values, calculated using a Benjamini and Hochberg correction. Corrected p-values of 0.05 were used as the cutoff for significance, corresponding to a significance threshold of |fold change|>1.4, adjusted p-value<0.05, using a moderated t-statistic (LIMMA). The microarray data for this study are available with accession number GSE45583 through NCBI’s Gene Expression Omnibus (GEO).

For qRT-PCR analysis, 1 µg of total RNA was reverse transcribed using iScript (BioRad) according to the manufacturer’s instructions. qPCR reactions were then performed using TaqMan Gene Expression Assays (Applied Biosystems; for primers used see Table S2) and an ABI7500 Fast Real-Time PCR System. Relative mRNA levels were calculated through comparison with GAPDH amplification values.

### Supplementary Information

Supplementary information is presented with this manuscript.

## Supporting Information

Figure S1 **Modeling of the location of the point mutations in the 3D structure of Lsd1.** Computer modeling of the structure of Lsd1 indicates that the point mutation at position 413 is present at the base of the tower domain, and may have effects on the structure of both the tower and the amine oxidase domain. The mutation at position 448 occurs at a residue that is known to be involved in binding to CoREST, and as such may affect Lsd1 protein-protein interactions.
(TIF)

Figure S2 **Analysis of heart development in the 2lox/2lox mice.** (A) BrdU incorporation into the hearts of E13.5 mice is similar between the wild-type and 2lox/2lox mice, indicating that the proliferation of cells in these hearts is not altered. (B) Staining of cardiomyocytes with sarcomere myosin antibody (MF20) demonstrates no lack of cell colonization of the septum in the 2lox/2lox hypomorphic hearts. No counterstain was used for the BrdU staining, and H&E was used as a counterstain for the MF20 staining.
(TIF)

Figure S3 **Lsd1 expression in MEF cell lines.** Protein expression of Lsd1 in MEFs demonstrates similar protein expression in heterozygous knockout and 2lox/2lox cells. The expression levels of Lsd1 (top panel) were examined in wild-type, heterozygous knockout (1lox/+) and homozygous hypomorphic (2lox/2lox) MEF lines. Tubulin served as a loading control (bottom panel).
(TIF)

Table S1 **PCR primers used for cloning,sequencing and mutagenesis in this study.**
(PDF)

Table S2 **Primers used for qPCR analysis in this study.**
(PDF)
