# Supplementary figures and images for: A Hypomorphic Lsd1 Allele Results in Heart Development Defects in Mice
Source: PLoS One. 2013 Apr 24;8(4):e60913. doi: 10.1371/journal.pone.0060913 (PMC3634827; doi:10.1371/journal.pone.0060913)

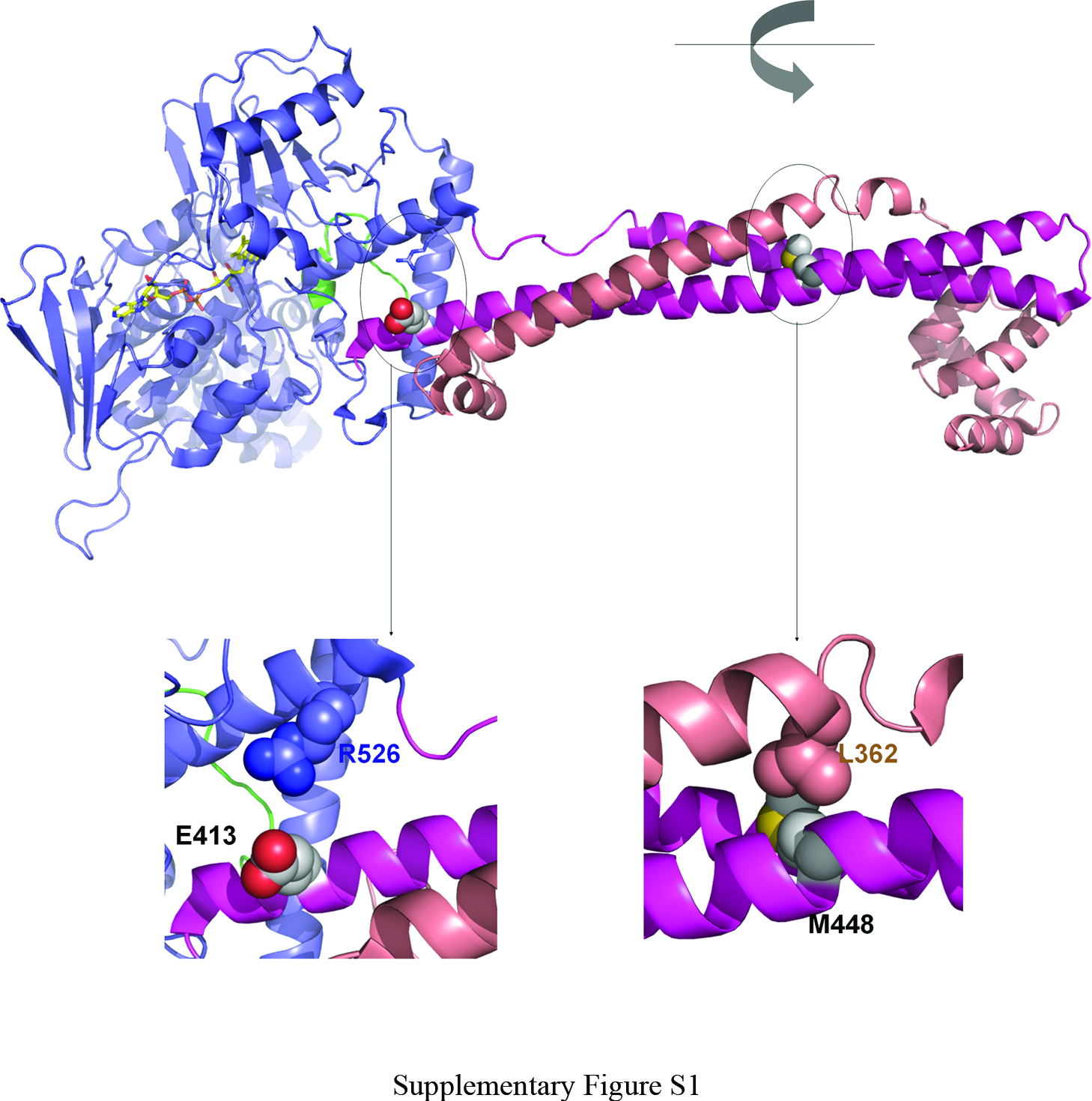

Supplement: Figure S1 — Modeling of the location of the point mutations in the 3D structure of Lsd1. Computer modeling of the structure of Lsd1 indicates that the point mutation at position 413 is present at the base of the tower domain, and may have effects on the structure of both the tower and the amine oxidase domain. The mutation at position 448 occurs at a residue that is known to be involved in binding to CoREST, and as such may affect Lsd1 protein-protein interactions. (TIF) [file pone.0060913.s001.tif]

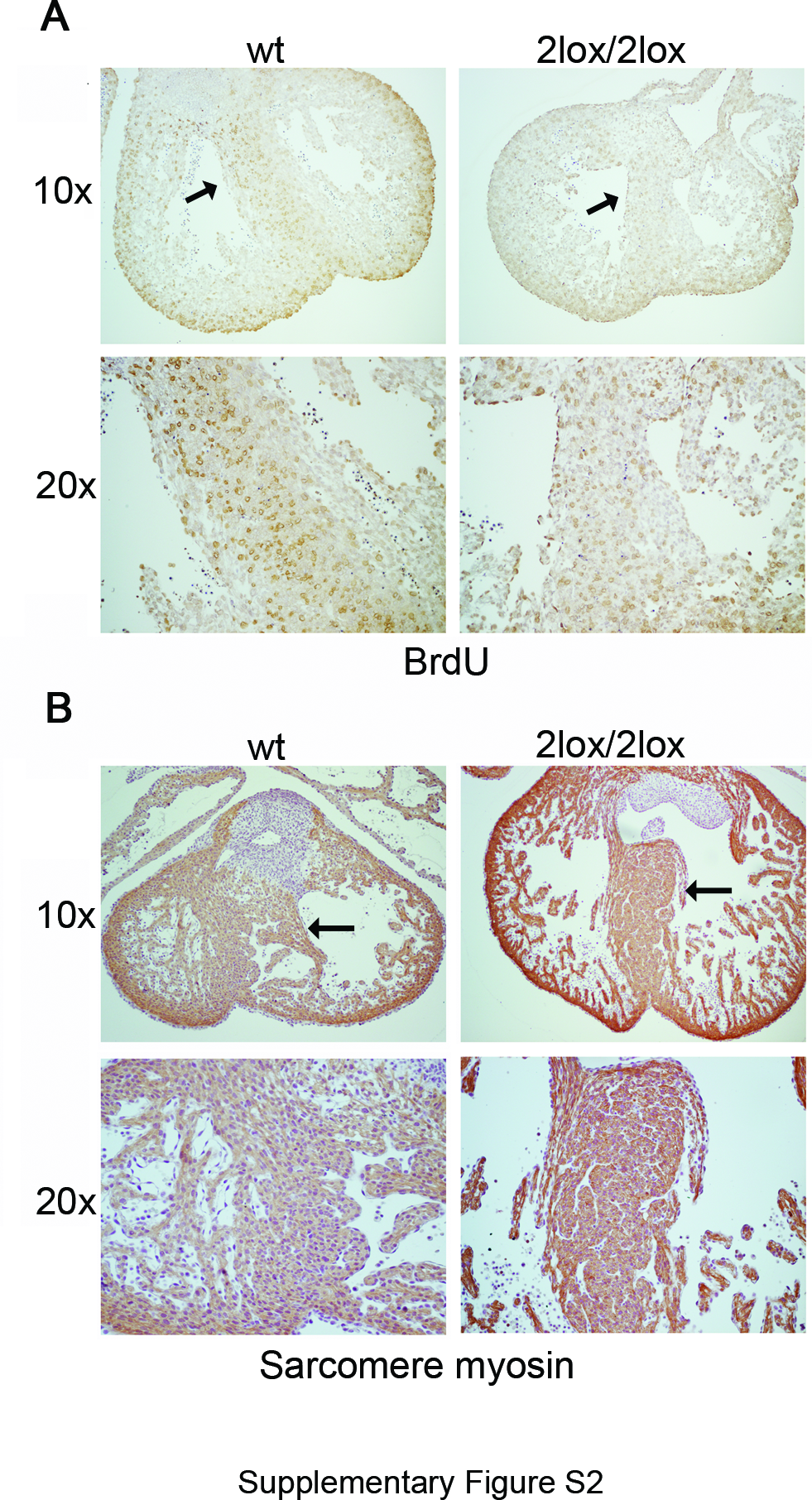

Supplement: Figure S2 — Analysis of heart development in the 2lox/2lox mice. (A) BrdU incorporation into the hearts of E13.5 mice is similar between the wild-type and 2lox/2lox mice, indicating that the proliferation of cells in these hearts is not altered. (B) Staining of cardiomyocytes with sarcomere myosin antibody (MF20) demonstrates no lack of cell colonization of the septum in the 2lox/2lox hypomorphic hearts. No counterstain was used for the BrdU staining, and H&E was used as a counterstain for the MF20 staining. (TIF) [file pone.0060913.s002.tif]

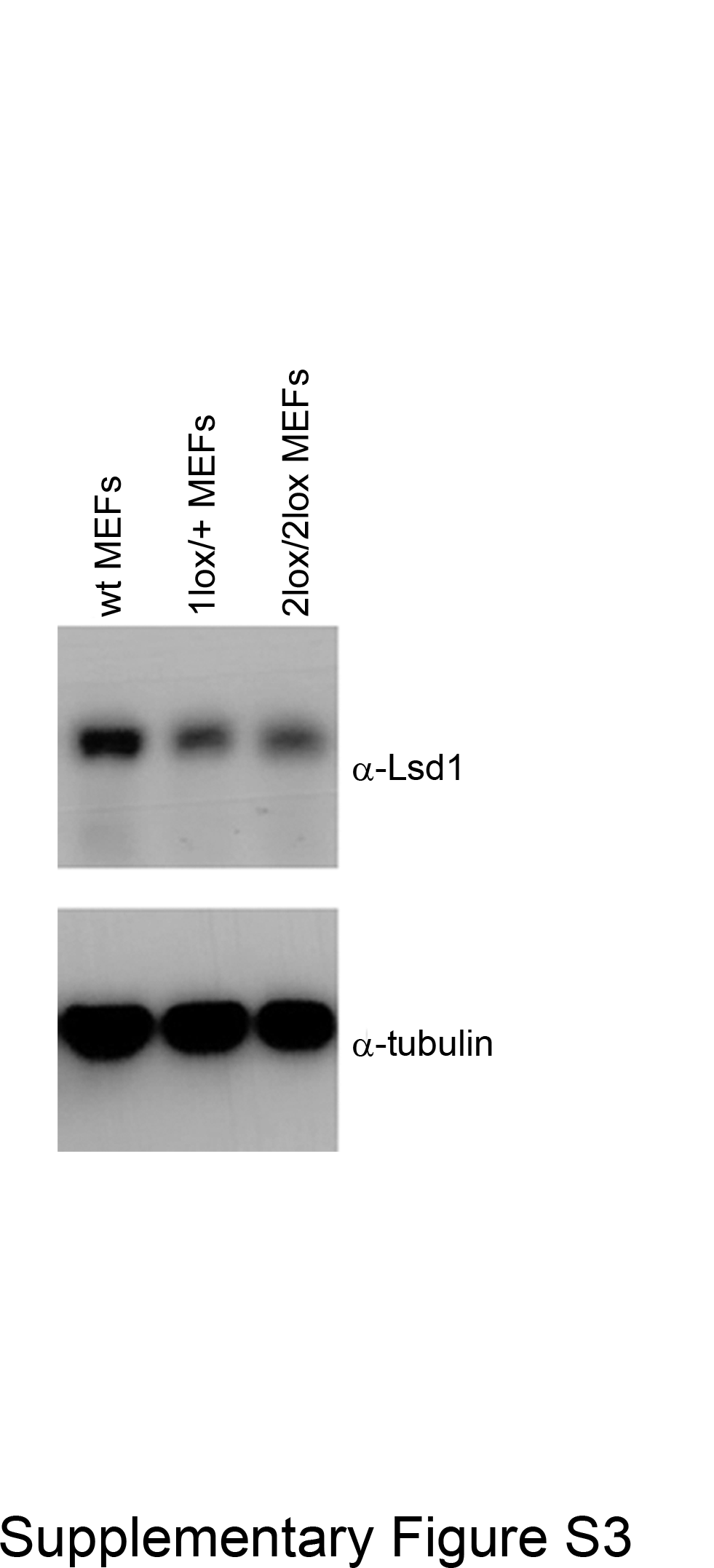

Supplement: Figure S3 — Lsd1 expression in MEF cell lines. Protein expression of Lsd1 in MEFs demonstrates similar protein expression in heterozygous knockout and 2lox/2lox cells. The expression levels of Lsd1 (top panel) were examined in wild-type, heterozygous knockout (1lox/+) and homozygous hypomorphic (2lox/2lox) MEF lines. Tubulin served as a loading control (bottom panel). (TIF) [file pone.0060913.s003.tif]
