# Supplementary material for: A Hypomorphic Lsd1 Allele Results in Heart Development Defects in Mice
Source: PLoS One. 2013 Apr 24;8(4):e60913. doi: 10.1371/journal.pone.0060913 (PMC3634827; doi:10.1371/journal.pone.0060913)
Supplement: Table S1 — PCR primers used for cloning,sequencing and mutagenesis in this study. (PDF) [file pone.0060913.s004.pdf]

Supplementary Table 1: PCR primers used for cloning,sequencing and mutagenesis in this study

cDNA	Direction	Sequence
LSD1	Forward	5'-GCGGAATTCGATGTTGTCTGGGAAGAAG
	Reverse	5'-ACCAGGTACCCTCACATACTTGGGGACTGC
N535A	Top	5'-GACAGACAAATACTTGACTGGCATTTTGCAGCTCTTGAATTCGCCAACGCCACACC
	Bottom	5'-GGTGTGGCGTTGGCGAATTCAAGAGCTGCAAAATGCCAGTCAAGTATTTGTCTGTC
E413G	Top	5'-GGCCAGGCATTGGGGGTTGTCATTCAGCTGC
	Bottom	5'-GCAGCTGAATGACAACCCCCAATGCCTGGCC
M448V	Top	5'-GAAAGAGCTTCTTAATAAGGTGGTAAATTTGAAGGAG
	Bottom	5'-CTCCTTCAAATTTACCACCTTATTAAGAAGCTCTTTC
LSD1 sequencing	forward 1*	5'-ACCATGGACTACAAAGACCATGACGG
	forward 2	5'-CGCACATTGCAGTTATGGCTGGAC
	forward 3	5'-CCGGTTGCTAGAAGCCACTTCTTACC
	forward 4	5'-CGCCACACCTCTCTCTACCCTC
	forward 5	5'-CCCTGGTAGCAGGAGAAGCTGC
	reverse 6*	5'-CCCGGGATCCTCACAGATCCTCTTC
	reverse 7	5'-CTGTGGGACTGCACTGCGC
	reverse 8	5'-GGTTTGACTTGTGGAACGTGTGTTCAC
	reverse 9	5'-TTTCAACTCCTCCTGAGTTTTCACTATCTCTTCC
	reverse 10	5'-GCCGAAGTTGATAAGACCATGGCGC

*: these sequences are complementary to the vector (p3XFLAG-myc-CMV-26) into which the cDNA was originally cloned
