# Supplementary material for: A Hypomorphic Lsd1 Allele Results in Heart Development Defects in Mice
Source: PLoS One. 2013 Apr 24;8(4):e60913. doi: 10.1371/journal.pone.0060913 (PMC3634827; doi:10.1371/journal.pone.0060913)
Supplement: Table S2 — Primers used for qPCR analysis in this study. (PDF) [file pone.0060913.s005.pdf]

Supplementary Table 2: Primers used for qPCR analysis in this study

Gene Symbol	Gene name	Applied Biosystems Assay ID
B4galt6	UDP-Gal:betaGlcNAc beta 1,4-galactosyltransferase, polypeptide 6	Mm00480045_m1
Camk2b	calcium/calmodulin-dependent protein kinase II beta	Mm00432284_m1
Cdh5	VE-cadherin	Mm03053719_s1
Fblim1	filamin binding LIM protein 1	Mm00505298_m1
Fn1	fibronectin 1	Mm01256744_m1
Gapdh	glyceraldehyde-3-phosphate dehydrogenase	Mm99999915_g1
Isl1	ISL1 transcription factor, LIM/homeodomain	Mm00517585_m1
Kdm1a	lysine-specific demethylase 1	Mm01181033_m1
Kit	kit oncogene	Mm00445212_m1
Lrp6	low density lipoprotein receptor-related protein 6	Mm00999795_m1
Ncam1	neural cell adhesion molecule 1	Mm01149710_m1
Nkx2-5	NK2 transcription factor related, locus 5	Mm00657783_m1
Pecam1	platelet/endothelial cell adhesion molecule 1	Mm01242584_m1
Tesc	tescalcin	Mm00498717_m1
Wnt11	wingless-related MMTV integration site 11	Mm00437328_m1
